# Income inequalities, social support and depressive symptoms among older adults in Europe: a multilevel cross-sectional study

**DOI:** 10.1007/s10433-021-00670-2

**Published:** 2021-12-17

**Authors:** Esteban Sánchez-Moreno, Lorena P. Gallardo-Peralta

**Affiliations:** 1 Department of Sociology, Methods and Theory, Research Institute in Development and Cooperation (IUDC-UCM), Universidad Complutense de Madrid, Donoso Cortés, 63, 28015 Madrid, Spain; 2 Dirección de Investigación, Postgrado y Transferencia Tecnológica (DIPTT), Universidad de Tarapacá, Arica, Chile

**Keywords:** Income inequality, Household income, Social support, Depression, Multilevel analysis

## Abstract

This study analysed the association between income inequality and depression from a multilevel perspective among older adults in Europe, including an examination of the role of social support. The data came from Eurostat’s European Health Interview Survey (EHIS). Selected participants were aged 65 years or above (*n* = 68,417) and located in 24 European countries. The outcome variable (depression) was measured using the eight-item Patient Health Questionnaire (PHQ-8). The resulting dataset included individual-level (level-1) and aggregate-level (level 2) exposure variables. Level-1 included income quintiles and social support as exposure variables and sex, age, living alone, limitation in activities of daily living and general activity limitation as control variables. Level 2 included the Gini coefficient, healthcare expenditure and dependency ratio. A multilevel linear regression analysis was performed with maximum likelihood (ML) estimation. All the income quintiles from 1 to 4 showed higher average scores for depression than quintile 5 (the highest). Higher social support scores were associated with lower scores for depression. An interaction was found between income quintile and social support, with higher levels of social support associated with lower scores for depression in quintiles 1 and 2. Higher Gini coefficient scores were associated with higher scores for depression. A significative random slope for social support was also found, meaning that the relationship between social support and depression differed across countries. No significant interaction was found between the Gini coefficient and social support. The study findings suggest that more unequal societies provide a less favourable context for the mental health of older adults. There are also significant country-dependent differences in terms of the relationship between support and mental health among older adults.

**Supplementary Information:**

The online version contains supplementary material available at 10.1007/s10433-021-00670-2.

## Introduction

Depression is a key issue during ageing. In Europe, depression is more prevalent among people aged over 65 years (and particularly among those over 75) than among other age groups (Arias-de-la-Torre et al. 2021). Depression decreases quality of life for older adults (Wels 2020), increases the risk of deterioration of health and functionality (Curran et al. 2020; Rodda et al. 2011) and is associated with a higher likelihood of loneliness (Domènech-Abella et al. 2020; Rokach 2019). The role of socioeconomic status (SES) in the production of different incidences of depression is convincingly supported by abundant empirical evidence (Lorant 2003). In the case of older adults, several recent studies in Europe have incorporated income into their analyses. The evidence generated by these studies suggests that household income inequality plays a remarkable role in the social gradient in depression among older adults (Brinda et al. 2016; Freeman et al. 2016; Hoebel et al. 2017). The income–depressive symptoms relationship among older adults is likely due to psychosocial factors (Geyer et al. 2006; Osafo et al. 2015). Low-income levels can create financial strain and daily stress, involving worries over financial matters and the future of household members (Zimmerman and Katon 2005). Moreover, the rank of a person’s income within a comparison group (for example, a region or country) may be associated with depression by acting as a proxy for actual and biographical social rank (Wood et al. 2012), insofar as individuals who perceive themselves as having a low social rank will be more likely to experience symptoms of depression.

Mental health among older adults may be associated with household income but also with the level of income inequality in a society. In the income inequality hypothesis (Wilkinson and Pickett 2007), the individual’s mental health is influenced by the degree of income inequality in the community/society. This hypothesis suggests that mental health at the individual level is affected by individual/household absolute income but also by relative income, deprivation and social status (Wagstaff and van Doorslaer 2000). Income inequality at the population level may accentuate the role of relative differences in these respects and hence have a negative impact on depression at the individual level. Meanwhile, egalitarian societies could improve individual mental health given their higher degree of social cohesion and social capital. These societies would be characterised by social relationships based on trust and belonging. In contrast, less equal societies could negatively impact in psychosocial processes that are particularly significant for mental health (self-esteem, interpersonal trust, social relationships) and negative processes of social comparison associated with feelings including inadequacy, shame and hostility (Wilkinson 1996). This line of research has generated particularly lively debate and enjoys reasonable empirical support (Chauvel and Leist 2015; Kawachi et al. 1997; Messias et al. 2011). The recent trends of rising inequality in Europe, where the scale and significance of income inequality have increased since the Great Recession of 2008 (Cohen and Ladaique 2018), pose the challenge of understanding how this growth in income inequality is affecting mental health at population level. Although it appears to be having a relatively lower impact for older adults in Europe (Caminada et al. 2017), rising income inequality remains relevant for this group because it implies a potential change in the effectiveness, generosity and level of coverage provided by social protection systems—particularly pension systems (Been et al. 2017)—and potential growth in medium- and long-term income inequality among older adults (Raitano 2016). An increased inequality gap might be associated with higher prevalence of symptoms of depression among older adults. However, as stated in the meta-analysis conducted by Patel et al. (2018), studies examining this issue in the specific case of older adults are generally scarce, particularly in Europe, and have produced mixed and inconclusive results.

The aim of this study is to contribute to an understanding of the association between household income, societal inequality of income and depression among older adults in Europe, using techniques that allow for an exploration of the role played by variables applying various levels of analysis. The study also aims to identify mechanisms that contribute to understanding this association in the specific case of older adults, incorporating into the analysis the concept of social support, a psychosocial process whose relationship with depression has been clearly established (Ayalon and Levkovich 2019; Harasemiw et al. 2019). Social support can be defined as emotional, material/instrumental and informational assistance received by significant members of one’s social network. It is particularly relevant in the case of older adults (Russell and Cutrona 1991), given that the empirical evidence has consistently supported its protective role in terms of mental health, both directly (through the benefits of social relationships) and as a buffer for the potential negative impact of stressful circumstances and the changes and adjustments that accompany the ageing process (Gariépy et al. 2016). This protective effect of social support suggests the existence of a moderating role with regard to the impact of household income and income inequalities on differences in the prevalence of symptoms of depression. As regards household income, the classic study by Cohen and Wills (1985) posited the buffering hypothesis—and its tension with the direct effects hypothesis—as a key factor in the debate on the role of social support in health. According to the buffering hypothesis, stressful situations will have a lower impact on mental health among people who have higher levels of social support. However, recent studies show that the proportion of studies that detect a buffering role for social support is significantly lower than those that report a direct association. In their systematic review, Tajvar et al. (2016) concluded that there was weak evidence for a stress-buffering role of social support from studies specifically carried out with older adults. The findings of a study by Russell and Cutrona (1991) had previously questioned the existence for this group of a buffering role against stressful events, suggesting instead that social support reduces the likelihood of experiencing daily hassles—that is, lower-intensity but constant and persistent day-to-day situations. Negative day-to-day experiences associated with low income might therefore have fewer consequences for mental health among older people with higher levels of social support (Lee and Chou, 2019). In addition, in relation to the income inequality hypothesis, Wilkinson and Pickett (2006) observed that more equal societies are characterised by the existence of high-quality relationships based on trust and belonging, generating support for individuals. In contrast, a trend towards greater social inequality is associated with less solidarity-based and more conflictive social relationships, as well as increased interpersonal hostility. These arguments support the hypothesis that as income inequality rises in a society, the levels of social support will fall among the population, contributing to increased levels of depression among older adults. The empirical evidence in this respect is scarce but suggests that it would be useful to explore this pathway in the contexts of household income (Cross-Denny and Robinson 2017) and the income inequality hypothesis (Haseda et al. 2018a).

Within this framework, two gaps can be identified in the existing literature on income inequality and depression among older adults. First, few studies have analysed the association between income inequality and depression among older adults in Europe from a multilevel perspective. Second, even fewer studies contain analyses that take into account the processes that might contribute to understanding the mechanisms of this association. In this vein, the scant importance accorded by previous research to the potential role of social support in the case of older adults is particularly striking. In order to contribute in these two areas, this study examined the following hypotheses:

1. Household income and social support: (a) will be directly and independently associated with scores for depression; and (b) will interact in a way that generates significant coefficients in the lower income quintiles.
2. A significant positive association is expected to be found between income inequality and depression: as income inequality increases, so will depressive symptoms.
3. The association between social support and depression will vary in intensity from country to country.
4. An interaction is expected to be found between social support and income inequality, whereby receiving social support diminishes the association between income inequality and depression.

## Materials and methods

### Data and participants

The data were taken from the European Health Interview Survey (EHIS) (Eurostat 2013) conducted by Eurostat, the European Commission’s statistical agency. This survey was designed to generate harmonised and comparable data for the European Union’s Member States. Participants aged 65 years or over were selected for this study (*n* = 68,417). The data have a nested two-level structure and are made up of 68,417 individuals located in 24 countries. The data were collected in 2014 and 2015, except for Austria and the UK (2013). Both of these countries were retained as part of the dataset owing to their interest in the regional context. The sample distribution by country and the main sociodemographic characteristics of the sample can be found in the supplementary material (supplementary table 1). Although the sample design included national variations, nationally representative probability samples were obtained in all cases. Also in all cases, the collection of data was spread over at least three months, including at least one month of the autumn season (September–December). The reported unit non-response exceeded 50% in Denmark, Germany, Luxembourg, Austria and Finland. The total item non-response (unweighted and before imputation) varied from country to country. At one extreme, the highest rates were found for Finland (16.8%) and Ireland (7.9%). At the other, the lowest rates were in the Czech Republic (0.1%) and Slovenia (0.1%). There were only three countries in which the non-response rate for any of the items corresponding to the outcome variable (depression) was higher than 10%. For the exposure variables, the non-response rate for a variable required to construct the “income” variable” exceeded 10% in Slovenia, Poland, Luxembourg, Denmark, Estonia, France and Hungary. In the case of the “social support” variable, a rate in excess of 10% was only recorded for one of the items comprising the scale (number 3) in three countries. Proxy interviews were used in cases where the respondent was unable to answer because they suffered from long-term cognitive impairment, long-term severe debilitation, long-term sensory impairment that prevented interaction, or were in hospital or a health or social care facility for the entire period of the fieldwork. Proxy interviews accounted for 3.8% of the total. Ethical approvals were obtained for the national surveys in accordance with the national guidance and regulation at the time of data collection, as well as with the terms specified by Eurostat. As a result, this study complies with the principles of the Declaration of Helsinki.

### Measures

#### Outcome measure

The eight-item Patient Health Questionnaire (PHQ-8) (Kroenke and Spitzer 2002) was used to assess depression. The PHQ-8 consists of eight of the nine criteria that provide the basis for the DSM-IV and V diagnosis of depressive disorders (little interest or pleasure, depressed mood, disturbed sleep, tiredness or little energy, poor appetite or overeating, feelings of worthlessness or guilt, having trouble concentrating, and psychomotor retardation or agitation within the last two weeks). Research indicates that the omission of suicidal or self-injurious thoughts only has a minor effect on scoring because thoughts of self-harm are fairly uncommon among the general population (Lee et al. 2007). The set responses were “not at all”, “several days”, “more than half the days” and “nearly every day”, with points assigned to each criterion accordingly (0 to 3, respectively). Following Kroenke et al. (2001), the sum score can be classified into four categories: no significant depressive symptoms (0–4), mild (5–9), moderate (10–14), moderately severe (15–19), and severe depressive symptoms (20–24). The PHQ-8 is useful and suitable for clinical use as well as for general population studies to screen for current depressive symptoms (Kroenke et al., 2009; Kroenke and Spitzer, 2002). In the present study, the internal consistency (Cronbach’s alpha) of the scale ranged from 0.70 (Norway) to 0.93 (Romania). A PHQ-8 sum score was used as the outcome variable.

#### Level-1 exposure measures

The classification of the population into the quintiles in which participants were located at the time of the survey, defined based on net monthly equivalised household income, was used to analyse the role of income. This variable was compiled using information regarding household income, size and composition. Measurement included total household income available for spending or saving after tax and other deductions. All monetary income received by each household member from any source was included: income from work (including self-employment), property and investment income (including pensions from individual private plans), and social benefits intended to help the household manage the financial burden of a number of risks or needs (unemployment, pension, survivor, sickness and disability benefits, and education, family/children and housing allowances), plus any other household income (for example, regular inter-household cash transfers). Any payments of taxes or social contributions were deducted. The result was divided by equalised household size, defined as a sum of weights attributed to each household member according to the modified Organisation for Economic Co-operation and Development (OECD) equivalence scale: 1.0 to the first adult; 0.5 to the second and each subsequent person aged 14 and over; 0.3 to each child aged under 14. For the conversion into quintile classes/groups, the data were ordered according to the value of the equalised total net monthly household income. Four cut-point values (quintiles) of income were found, dividing the survey population into five groups, each equally represented by 20% of respondents. This strategy incorporates stratification of income and not merely its distribution among the population. Participants were categorised into income quintiles based upon their ranking in each country.

Social support was measured using the Oslo-3 Social Support Scale (OSSS-3) with three questions (Meltzer 2003). The response categories were assessed independently for each of the three questions, and a sum score was created. The OSSS-3 has been used in several studies, confirming its feasibility and predictive validity with respect to psychological distress. The scale items are as follows: “How many people are you so close to that you can count on them if you have great personal problems?” [none (1), 1–2 (2), 3–5 (3), 5 + (4)]; “How much interest and concern do people show in what you do?” [a lot (5), some (4), uncertain (3), little (2), none (1)]; and “How easy is it to get practical help from neighbours if you should need it?” [very easy (5), easy (4), possible (3), difficult (2), very difficult (1)]. The sum score ranges from 3 to 14, with high values representing strong levels of social support and low values representing poor levels. This continuous score was used to generate the normative data for the OSSS-3 for each scoring point. Following Bøen et al. (2012), the sum score can be operationalised into three broad categories of social support: poor (3–8), moderate (9–11) and strong social support (12–14). There is evidence supporting the reliability and validity of the OSSS-3 as a measure of social determinants of health among the general population (Kocalevent 2018), as well as its applicability to different cultural contexts and age groups, including older adults (Bøen et al. 2012).

#### Level-1 control variables

Six control variables were incorporated into all the statistical models, all with particular significance in the literature on differences in wellbeing and mental health among older adults. These variables were sex (1 = female), age, living alone, education, limitations on activities of daily living (ADL) and general activity limitation. ADL was evaluated through a number of questions measuring performance of the main usual activities (feeding oneself; getting in and out of bed or chairs; dressing and undressing; using toilets; bathing or showering) according to the International Classification of Functioning, Disability and Health (ICF). These activities are considered important for quality of life and in terms of level of autonomy. They influence the decision-making process concerning the choice between institutionalisation and remaining in the community. The EHIS survey asked: “Do you usually have difficulty doing any of these activities by yourself?” The answers were: no difficulty; yes, some difficulty; yes, a lot of difficulty; and I can’t achieve it by myself. The scores obtained for each activity were added together in the current study, producing a quantitative variable. In order to identify the specific role of income, educational level was included (below lower secondary; upper secondary and post-secondary non-tertiary; tertiary) as a control variable related to SES (Schnittker 2004).

General activity limitation was evaluated though the Global Activity Limitation Indicator (GALI) (Robine and Jagger 2003). The GALI consists of a single item (“For at least the last six months, have you been limited because of a health problem in activities people usually do?”) with three response categories (yes, strongly limited; yes, limited; no, not limited). GALI is a useful and valid instrument to assess global activity limitation in both health and non-health surveys (van Oyen et al. 2006).

#### Level-2 exposure measures

The Gini coefficient was included as a measure of socioeconomic inequality; it measures the deviation of income distribution among households within a country from a perfectly equal distribution. A value of 0 represents absolute equality, and a value of 1 represents absolute inequality. This measure is widely used in studies on income inequality (Milanovic and Roemer 2016) and specifically in the health area (Pickett and Wilkinson 2015). For this reason and owing to its suitability in terms of the main aim of this research (analysis of income inequalities and their relationship with social support as significant factors in depression), the Gini coefficient was selected over other measures such as poverty and unemployment. Additionally, two level-2 control variables were incorporated. First, healthcare expenditure (expenditure allocated to treating illness, healthcare and disability) in each country as a percentage of gross domestic product (GDP) was included, given the available empirical evidence regarding its association with various health indicators (Nixon and Ulmann 2006). Second, the dependency ratio (defined as the ratio of the population aged 65 and over to the population aged from 15 to 64 years) was included, in order to control variability between countries in terms of population ageing, given the role it has played in previous research concerning differences in depression (Arias-de-la-Torre et al. 2021).

### Analytical strategy

Given the nested structure of the data, a linear multilevel regression analysis was performed with maximum likelihood (ML) estimation. Multilevel regression analysis provides an analytical framework when the processes that affect the outcome variable are hypothesised to operate at more than one level. Its use offers two fundamental advantages (West et al. 2015). First, multilevel analysis permits analysis in the same model of the impact of variables corresponding to different levels, including multilevel interaction. Second, it facilitates the incorporation of fixed and random effects into the specification of the model.

Multilevel techniques provide estimates of relationships between individual variables and the variations between countries that cannot be explained by such variables. Moreover, it is possible to estimate the variation in selected individual associations between countries (random-slope parameters) and, at the same time, the interaction between individual-level variables and contextual-level measures. ML estimation is generally robust, producing estimates that are asymptotically efficient and consistent. In fact, with large samples as in the case of this study, ML estimates are robust against mild violations of the assumptions (Maas and Hox 2004). In order to reduce the risks of multicollinearity and facilitate the interpretation of the estimators, the exposure variables were grand-mean centred. The unstructured covariance structure model was used to fit the data for the models including a random slope. This structure is the most parsimonious and requires no assumption in the error structure (Shek and Ma 2011). An unstructured covariance matrix permits estimation of the intercept and slope variances when the latter is included in the random part of the model, in addition to the covariance between the intercept and slope. The necessity to detect both fixed and random effects coefficients increases complexity in terms of power and sample size issues in multilevel linear models. Recommendations by Kreft and de Leew (1998) suggest that if the model incorporates cross-level interactions (as in the case at hand), then the number of clusters must be 20 or more in the higher level variables, in addition to having adequate group sizes. The multilevel structure for the data used in this study is made up of 68,417 individuals (level-1) nested within 24 countries (level 2). The minimum *n* corresponds to Luxembourg (*n* = 474). Accordingly, our dataset was aligned with the aforementioned recommendations.

Following the recommendation of Hox (2010 p. 56), an analysis was conducted taking the simplest model as a starting point and subsequently introducing the various parameters on a step-by-step basis. The analysis therefore started with the fixed parameters and ended with the variance components in five nested models. Model 1 (hypothesis 1a) included only the level-1 fixed effects parameters. Model 2 (hypothesis 1b) incorporated the interaction between social support and income quintiles. In both of these cases, the analysis only included random intercept. As a whole, models 1 and 2 analyse the role played by inter-individual differences in social support and income. Model 3 (hypothesis 2) adds parameters corresponding to level-2 variable fixed effects (Gini coefficient, healthcare expenditure and dependency ratio). The random slope corresponding to the social support variable was incorporated into model 4 (hypothesis 3). Finally, model 5 (hypothesis 4, full model) included the cross-level interactions between social support (level-1) and the Gini coefficient (level 2).

The general design for the full model tested in our work is the following:

$$PHQ_{{{\text{ij}}}} = \gamma_{00} + \gamma_{{{\text{p}}0}} X_{{{\text{pij}}}} + \gamma_{{0{\text{q}}}} Z_{{{\text{qj}}}} + \gamma_{{{\text{pq}}}} X_{{{\text{ij}}}} Z_{{\text{j}}} + \mu_{{{\text{pj}}}} X_{{{\text{pij}}}} + \mu_{{0{\text{j}}}} + e_{{{\text{ij}}}}$$

where PHQ-8 of individual *i* in country *j* is a function of p level-1 variables ($X_{{{\text{pij}}}}$) and q level-2 (country) variables ($Z_{{{\text{qj}}}}$). Cross-level interactions are included ($\gamma_{{{\text{pq}}}} X_{{{\text{ij}}}} Z_{{\text{j}}}$). Regression intercept ($\gamma_{00}$), random slopes ($\mu_{{{\text{pj}}}} X_{{{\text{pij}}}}$) and error terms ($\mu_{{0{\text{j}}}} + e_{{{\text{ij}}}}$) are reported. The full model is designed as follows:

$$\begin{aligned} {\text{PHQ}}_{{{\text{ijt}}}} & = \gamma_{00} + \gamma_{10} X_{{1{\text{ij}}}} + \gamma_{20} X_{{2{\text{ij}}}} + \gamma_{30} X_{{3{\text{ij}}}} + \gamma_{40} X_{{4{\text{ij}}}} + \gamma_{50} X_{{5{\text{ij}}}} \\ & \quad + \gamma_{60} X_{{6{\text{ij}}}} + \gamma_{70} X_{{7{\text{ij}}}} + \gamma_{80} X_{{8{\text{ij}}}} + \gamma_{90} X_{{9{\text{ij}}}} + \gamma_{100} X_{{7{\text{ij}}}} X_{{8{\text{ij}}}} { } \\ & \quad + \gamma_{01} Z_{{1{\text{j}}}} + \gamma_{02} Z_{{2{\text{j}}}} + \gamma_{03} Z_{{3{\text{j}}}} + \gamma_{83} X_{{8{\text{ij}}}} Z_{{3{\text{j}}}} + \mu_{{8{\text{j}}}} X_{{8{\text{ij}}}} + \mu_{{0{\text{j}}}} + e_{{{\text{ij}}}} \\ \end{aligned}$$

For level-1 variables, $X_{{1{\text{ij}}}}$ represents the “age” variable, $X_{{2{\text{ij}}}}$ is “sex (female)”, $X_{{3{\text{ij}}}}$ is “living alone”, $X_{{4{\text{ij}}}}$ is “GALI”, $X_{{5{\text{ij}}}}$ is “limitation in ADL”, $X_{{6{\text{ij}}}}$ is “education”, $X_{{7{\text{ij}}}}$ is “income quintile”, and $X_{{8{\text{ij}}}}$ is “social support”. For level-2 variables, $Z_{{1{\text{j}}}}$ represents “dependency ratio”, $Z_{{2{\text{j}}}}$ is “Gini coefficient”, and $Z_{{3{\text{j}}}}$ is “healthcare expenditure”. For interactions, $X_{{7{\text{ij}}}} X_{{8{\text{ij}}}}$ represents the interaction between “income quintile” and “social support” (level-1 interaction) and $X_{{8{\text{ij}}}} Z_{{3{\text{j}}}}$ represents the interaction between “social support” and “Gini coefficient” (cross-level interaction). The random slope for “social support” is $\mu_{{8{\text{j}}}} X_{{8{\text{ij}}}}$.

The robustness of the main findings to alternative variance specifications was assessed via a bootstrapping analysis (random sampling). Bootstrapping is a widely used method for evaluating the uncertainty associated with a given estimator. Random samples of the dataset are taken, model specifications are run on each random sample, and a 95% bootstrap confidence interval for the primary finding is generated. The analyses were performed using IBM-SPSS V25.

## Results

Table 1 shows the descriptive results for the main level-1 and level-2 study variables by country. As can be observed, the average scores for the PHQ-8 and OSSS-3 scales varied significantly across the different countries included in the study. These results supported the design of the analysis and specifically the incorporation of random coefficients for the intercept and slope of social support (OSSS-3).

**Table 1:** Descriptive results for the main level-1 (individual) and level-2 (country) study variables

	Level-1 variables Mean (SD)			Level-2 variables Value		
	PHQ-8—summatory	Social support—summatory	Activities of daily living—summatory	Gini coefficient	Healthcare expenditure	Dependency ratio
Austria	2.55 (.06)	10.99 (.04)	0.32 (.02)	.276	9.30	27.20
Bulgaria	4.81 (.15)	9.87 (.05)	1.55 (.08)	.354	6.30	29.30
Czechia	2.84 (.07)	10.34 (.04)	1.33 (.05)	.251	7.20	25.70
Germany	3.14 (.05)	10.22 (.03)	0.69 (.03)	.301	12.10	32.00
Denmark	2.13 (.09)	9.93 (.05)	0.35 (.04)	.274	12.00	28.80
Estonia	4.09 (.11)	9.84 (.05)	0.62 (.05)	.356	6.00	27.90
Greece	2.79 (.08)	9.68 (.04)	1.52 (.06)	.345	6.30	31.60
Finland	1.91 (.08)	9.67 (.05)	0.56 (.04)	.256	10.80	30.20
France	3.36 (.08)	9.84 (.04)	0.65 (.04)	.292	11.20	28.30
Hungary	5.11 (.14)	10.55 (.06)	1.24 (.07)	.286	6.30	25.80
Ireland	2.40 (.08)	10.96 (.04)	0.66 (.04)	.297	6.30	19.70
Italy	3.95 (.06)	10.19 (.02)	1.37 (.04)	.324	8.40	33.10
Lithuania	3.32 (.11)	10.22 (.05)	1.48 (.08)	.350	5.50	27.50
Luxembourg	3.30 (.16)	10.09 (.09)	0.47 (.07)	.287	8.10	20.40
Latvia	3.17 (.09)	9.53 (.05)	1.18 (.06)	.355	4.80	28.80
Norway	1.85 (.06)	11.02 (.04)	0.15 (.02)	.239	12.80	24.50
Poland	3.93 (.06)	10.33 (.03)	1.32 (.03)	.308	5.90	21.20
Portugal	4.66 (.07)	10.59 (.03)	0.80 (.03)	.345	8.00	30.30
Romania	4.71 (.08)	9.33 (.03)	1.06 (.03)	.350	5.00	24.30
Sweden	2.17 (.11)	10.51 (.06)	0.48 (.05)	.269	10.60	30.60
Slovenia	2.90 (.10)	10.92 (.05)	0.94 (.06)	.250	8.70	25.70
Slovakia	3.27 (.11)	9.22 (.06)	1.44 (.07)	.261	7.20	19.00
UK	2.15 (.04)	11.44 (.03)	0.50 (.02)	.302	10.30	26.40
Iceland	3.04 (.14)	11.86 (.06)	0.41 (.05)	.247	11.50	20.50

Table 2 shows the results obtained in the multilevel regression analysis for the five models that were produced. The results corresponding to fixed coefficients are shown first. Social support and household income (level-1 variables) can be observed to have been significantly associated with depression. All the income quintiles from 1 to 4 showed higher average scores than quintile 5 (the highest, reference quintile). The size of the association increased as income fell, such that the coefficient for the first quintile was $\gamma$=0.37 (*p* < 0.001), while for the fourth quintile it was $\gamma$=0.16 (*p* < 0.01). Additionally, higher social support scores were associated with lower scores for depression across all the models (${\gamma }_{80}$= −0.16; *p* < 0.001). Moreover, the interaction between income and social support resulted in statistically significant coefficients across all models, with high levels of social support related to lower scores for depression for the lowest-income quintile ($\gamma$= −0.11; *p* < 0.001) and, to a lesser extent, for the second quintile ($\gamma$= -0.07; *p* < 0.01), taking the highest-income quintile as a reference (Q5). As regards level-2 variables, higher Gini coefficient scores were associated with higher scores for depression (${\gamma }_{02}$=15.41; *p* < 0.05), bearing in mind that the inclusion of a random effect of social support in model 4 resulted in a slightly lower coefficient for the Gini measure (${\gamma }_{02}$=0.11.40; *p* < 0.05). Of the level-2 control variables, only healthcare expenditure was associated with depression.

**Table 2:** Multilevel estimated coefficients, standard error and confidence interval (95%)^a^ of country-level and individual-level variables on depression (PHQ-8)

Variable	Lvl	Model 1	Model 2	Model 3	Model 4	Model 5
Fixed effects						
Intercept	1	2.13*** (.18) [1.76,2.50]	2.11*** (.18) [1.73,2.48]	2.19*** (.18) [1.81,2.58]	2.21*** (.18) [1.84,2.59]	2.23*** (.18) [1.85,2.61]
Age	1	.07** (.03) [.01,.14]	.08** (.03) [.01,.15]	.08* (.03) [.01,.14]	.08* (.03) [.01,.14]	.08* (.03) [.01,.14]
Female	1	− .69*** (.03) [− .75, − .62]	− .69*** (.03) [− .75, − .62]	− .68*** (.03) [− .75, − .63]	− .69*** (.03) [− .75, − .62]	− .69*** (.03) [− .75, − .62]
Living alone	1	.12*** (.03) [.05,.18]	.12*** (.03) [.05,.18]	.12*** (.03) [.05,.18]	.12*** (.03) [.05,.18]	.12*** (.03) [.05,.18]
GALI	1					
* 1 (Limited but not severely)*		3.28*** (.05) [3.17,3.39]	3.28*** (.05) [3.17,3.39]	3.28*** (.05) [3.17,3.39]	3.28*** (.05) [3.17,3.39]	3.28*** (.05) [3.17,3.39]
* 2 (Severely limited)*		1.39*** (.03) [1.33,1.46]	1.40*** (.03) [1.33,1.46]	1.40*** (.03) [1.33,1.46]	1.40*** (.03) [1.33,1.46]	1.40*** (.03) [1.33,1.46]
ADL	1	.75*** (.008) [.73,.77]	.75*** (.008) [.73,.77]	.75*** (.008) [.73,.77]	.75*** (.008) [.73,.77]	.75*** (.008) [.73,.77]
Education (ref. University)	1					
* Compulsory*		.18*** (.05) [.08,.28]	.19*** (.05) [.09,.28]	.19*** (.05) [.09,.28]	.19*** (.05) [.09,.29]	.19*** (.05) [.09,.29]
* Secondary*		− .02 (.04) [− .11,.07]	− .01 (.04) [− .10,.08]	− .01 (.04) [− .10,.08]	− .01 (.04) [− .10,.08]	− .01 (.04) [− .10,.08]
Income Quintile (ref. Q5, highest income*)*	1					
* Q1*		.36*** (.05) [.25,.48]	.37*** (.05) [.26,.49]	.37*** (.05) [.26,.49]	.37*** (.05) [.25,.49]	.37*** (.05) [.25,.49]
* Q2*		.22*** (.05) [.12,.33]	.24*** (.05) [.13,.35]	.24*** (.05) [.13,.35]	.24*** (.05) [.13,.34]	.24*** (.05) [.13,.34]
* Q3*		.22*** (.05) [.11,.32]	.23*** (.05) [.12,.34]	.23*** (.05) [.12,.34]	.23*** (.05) [.12,.33]	.22*** (.05) [.12,.33]
* Q4*		.16** (.05) [.05,.27]	.17** (.05) [.06,.28]	.17** (.05) [.06,.28]	.16** (.05) [.05,.28]	.16** (.05) [.05,.28]
Social support	1	− .21*** (.007) [− .23, − .20]	− .16*** (.02) [− .20, − .12]	− .16*** (.02) [− .20, − .12]	− .16*** (.02) [− .21, − .11]	− .16*** (.02) [− .21, − .11]
Gini coefficient	2			15.41* (7.39) [− .05,30.89]	11.10* (5.12) [.37,21.84]	13.51* (6.03) [1.06,25.97]
Dependency ratio	2			− .08 (.05) [− .19,.03]	− .02 (.03) [− .10,.05]	− .03 (.03) [− .11,.05]
Healthcare expenditure	2			.16 (.11) [− .06,.41]	.17* (.07) [.008,.32]	.17* (.07) [.008,.34]
Interactions						
Social support * Income quintile	1 × 1					
* Q1*			− .11*** (.02) [− .17, − .06]	− .11*** (.02) [− .17, − .06]	− .11*** (.02) [− .16, − .05]	− .11*** (.02) [− .16, − .05]
* Q2*			− .07** (.02) [− .13, − .02]	− .07** (.02) [− .13, − .02]	− .07** (.02) [− .12, − .02]	− .07** (.02) [− .12, − .02]
* Q3*			− .01 (.02) [− .06,.03]	− .01 (.02) [− .06,.03]	− .01 (.02) [− .06,.03]	− .01 (.02) [− .06,.03]
* Q4*			− .02 (.02) [− .07,.03]	− .02 (.02) [− .07,.03]	− .01 (.02) [− .07,.03]	− .01 (.02) [− .07,.03]
Gini * social support	2 × 1					− .27 (.34) [− .98,.44]
Random effects						
Residual		11.03*** (.07) [10.88,11.17]	11.01*** (.07) [10.88,11.16]	11.01*** (.07) [10.87,11.15]	11.01*** (.07) [10.87,11.15]	11.00*** (.07) [10.86,11.14]
Intercept		.68** (.20) [.37,1.23]	.67** (.20) [.37,1.23]	.64** (.21) [.33,1.22]	.63** (.20) [.33,1.19]	.63** (.21) [.33,1.20]
Social support					− .03**(.01) [− .06, − .01]	− .03*(.01) [− .06, − .007]
Intercept*social support					.002*(.001) [.0008, .005]	.002*(.001) [.0007, .005]
Model fit						
Log.likelihood-2		246,049.36	246,037.49*	246,035.27	246,008.19***	246,007.89
Akaike (AIC)		246,053.36	246,041.49*	246,039.27	246,016.19***	246,015.89

^a^ Format: Multilevel coefficient (standard error) [95% confidence interval]

***p < .001; **p < .01; * *p* < .05

Model 4 incorporated the random coefficient for the social support measure into the equation, resulting in significant coefficients for both the variance of the random slopes (${\mu }_{8\mathrm{j}}$= −0.03; *p* < 0.01) and its covariance with intercepts [Cov(${\mu }_{0\mathrm{j}}$,${\mu }_{8\mathrm{j}}$) = 0.002; *p* < 0.05]. The first of these findings shows that the intercepts corresponding to social support in the regression equations across the different countries were not the same. Their value indicates the extent to which the country-specific slopes vary around a single slope fitted to the entire data (the overall slope, ${\gamma }_{80}$= − 0.16 in model 4). This means that the relationship between social support and depression scores widely differed across the countries considered in the sample. Following Snijders and Bosker (2012), the square root of the value of the random slope for social support can be interpreted as a measure of the standard deviation of the average slope (the fixed coefficient, ${\gamma }_{80}$= −0.16; *p* < 0.00), such that 95% of countries would have slopes for social support between 0.01 and −0.33. Additionally, the finding of a significant value [Cov(${\mu }_{0\mathrm{j}}$,${\mu }_{8\mathrm{j}}$) = 0.002; *p* < 0.05] for covariance between the value of the random slope for social support and the random intercept in the model implied that across countries, as the intercept value rises (that is, as average scores for depression in the countries rise), so does the size of the association between social support and depression. This means that in countries with higher average scores for depression, the association between social support and depression is more intense. Although the size of this value is small, it is worth noting that it is non-standardised. Finally, it should be noted that model 5, which incorporated cross-level interactions, did not result in significant values of improved goodness-of-fit for model 4.

To confirm the robustness of the findings, the results obtained for model 4 were further validated by bootstrapping analysis (random sampling). Analyses were also conducted to determine whether our results were driven by sex. The results are shown in the supplementary material (supplementary table 2). For all the variables included in the model, the 95% bootstrap confidence intervals obtained in the analysis replicate the results obtained in the original analysis. Taken as a whole, these results suggest stability and consistency in the findings obtained in this study.

## Discussion

Our results add to the empirical evidence supporting the argument that there is an association between SES and depression. First, differences in household income (measures based on income quintile) were related to significant differences in PHQ-8 scores (hypothesis 1a), insofar as the scores for the depression variable increased among the lower income quintiles. These results are aligned with those obtained in numerous previous studies (Domènech-Abella et al. 2018; Wilkinson 2016) and show the importance of considering the role of household income in understanding depression among older adults in Europe. Second, the positive association between the Gini coefficient and PHQ-8 scores suggests that income inequality (hypothesis 2) is a significant factor for older adults in European countries, in line with results obtained in other regions (Choi et al. 2015; Feng et al. 2012; Fernández Niño et al. 2014; Muramatsu 2003). This finding suggests that symptoms of depression are more common among older adults who are living in more unequal societies. This association between Gini coefficient and PHQ-8 scores is of remarkable significance given the scarcity of studies addressing this aspect (Marshall et al. 2014; Wu et al. 2020) and the mixed results obtained (Behanova et al. 2017). In European countries, Welfare States have acted to ensure that inequality gaps have remained at acceptable levels for decades. However, recent studies show that socioeconomic inequalities have significantly increased in recent years, with inequality gaps increasing from country to country and among the various social groups within each country (Eurofund 2017). Our results indicate that this phenomenon may be playing a role in the deterioration of mental health among older adults. Studies do suggest that the increase in inequality has been less intense for older adults (Benczur et al. 2017), given that two of the fundamental pillars of Welfare States are caring for older adults (particularly through the pension system) and the healthcare system (which is particularly important for older adults). However, the mental health of older adults may be affected by deteriorations in social relationships and interpersonal trust within a context of growing inequality that accentuates differences in SES (Pickett & Wilkinson, 2015). Additionally, more social inequality may increase the likelihood of a vicarious experience of stress and socioeconomic strain (Carnevali et al. 2020) among older adults. Even assuming that the effects of the growth of social inequalities on living conditions and SES in Europe are lower for this group than for younger generations, the increased socioeconomic difficulties experienced among the young (particularly adult children) within the family and/or in the households of older adults may be related to the latter groups feeling more worry, hopelessness, frustration or despondency (among other symptoms of depression).

The study findings suggest that social support plays a triple role. First, it has a direct association with PHQ-8 scores (hypothesis 1a), which is consistent with the existing research regarding the hypothesis of direct effects of social support (Wenger, 1997). Second, its interaction with income quintile (hypothesis 1b) suggests that social support might moderate the association between household income and depression. This result, which would be consistent with the discussion of the buffering hypothesis developed in the introduction to this article, refers exclusively to the two lowest income quintiles, suggesting that social support might be particularly significant for mental health among the most socioeconomically disadvantaged groups. For older adults, the situations of material deprivation associated with lower income quintiles may be associated less with stressful life events and more with daily hassles, shaping a range of daily experiences such that they have a lower impact on mental health for people who have higher levels of social support (Lee and Chou, 2019). Along these lines, numerous studies indicate that the use of networks of kin and other close relationships (mostly with neighbours and friends) constitutes a common strategy in the case of groups in economically disadvantaged situations (Lubbers et al. 2020a). These strategies are particularly significant for mental health, meaning that properly functioning social networks are a protective factor that reduces the likelihood of experiencing depression, anxiety (Lubbers et al. 2020b) and loneliness (Coll-Planas et al. 2017).

Third, the coefficient corresponding to the cross-level interaction between social support and income inequality (Gini coefficient) did not show a significant association with scores for depression (hypothesis 4). Available theoretical models maintain that societal inequality will have an impact on depression via psychosocial processes that include social capital, community insecurity and stress, giving rise to a situation of relative deprivation that is negative for the mental health of the population in general (Subramanian et al. 2003) and of older adults in particular (Ladin et al. 2010). These processes include and arise out of a deterioration in social relationships, and social support is therefore one of the psychosocial processes that might contribute to explaining the association between income inequality at the societal level and the prevalence of symptoms of depression (Haseda et al. 2018a, b). However, there are few studies in this respect in Europe. A study by González et al. (2020) analysed self-rated health scores for a cross-sectional sample with a nested structure from 30 countries in the International Social Survey Programme (ISSP) social networks survey module (2017). Applying a multilevel regression analysis (random intercept model), a significant interaction was found between the Gini coefficient and the social support measure, such that the greater the income gap, the weaker the association between social support and health.

However, the current study failed to find an interaction between social support and the Gini coefficient. This might be related to the confirmation of hypothesis 3, according to which the association between social support and depression varies significantly from country to country. This hypothesis was confirmed, with a significant coefficient obtained for the random slope corresponding to social support. Moreover, our results showed a significative covariance between social support random slopes and the random intercept (model 4), indicating that in countries with higher average scores for depression, the intensity of the association between social support and depression is also higher. The incorporation into the model of a random coefficient for social support constitutes a departure from the existing literature. In fact, to the best of our knowledge, this study is the first to address the potential moderating role of social support by including a fixed coefficient and a random coefficient in the model at the same time. The results obtained fit with recent theoretical approaches that emphasise the cultural complexity of the processes involved in receiving and perceiving support. As stated by Thoits (2011), social support implies action by significant others: people who form part of primary groups and comprise a fundamental source of support, particularly in emotional terms. It involves relationships based on the construction of intimate interpersonal ties (Brown and Harris 1978), which for older adults makes relationships with partner and friends particularly significant (Gariépy et al. 2016). However, the mobilisation and receipt of social support also includes action by similar others, who do not necessarily form part of primary groups. To the contrary, the relationship established with these similar others is based not so much on confidence but on the existence of shared experiences (Thoits 2020). In the case of older adults, this group would include neighbours and people participating in secondary and/or community groups created on the basis of experiences of the ageing process. As a result of the process of narrowing of the family network described in the literature (Greenfield 2012), these similarity-based ties can give rise to intimate relationships. The particularities of the surrounding social and cultural context are of particular importance in both cases. Processes involving establishing and developing relationships of confidence are strongly influenced by the cultural construction of the idea of intimacy, as well as by the specific social rules that guide social relationships in general, and families and friendship in particular. Moreover, relationships with similar others—especially those involving support—include interaction patterns (role-modelling, empathic understanding, advice) that are clearly influenced by sociocultural elements. In both cases, these processes of cultural construction necessarily entail notable variations depending on the country and society, which can create variations in the intensity and nature of the association between social support and depression across the different European countries. Our hypothesis 4 proposed that this variation could be understood partly through the interaction of social support with income inequality levels. The study findings did not support this proposal, pointing to a need to seek other processes that might help in understanding the differences in the association between social support and depression across different contexts and countries.

This study was subject to a number of limitations. First, the cross-sectional design does not allow for an evaluation of the impact of the time people have spent exposed to a context of low income (individual level) and/or income inequality (aggregate level), as it is impossible to establish a causal relationship between these processes and the measures in the PHQ-8. Second, the measure used for income (household in quintiles) may not take into account differences in individual circumstances, especially in cases of older adults who provide financial support to other relatives in situations of socioeconomic deprivation, and particularly their adult children. Third, the PHQ-8 is a screening instrument that assesses current depressive symptoms but does not provide a diagnosis of depression. Although the PHQ-8 is a widely used strategy in international health research, the reliability of responses can nonetheless be a matter of concern. The question here is whether or not there is major measurement error as a result of biased responses, owing to misunderstanding of questions or memory lapses. It is also necessary to note that different populations may use different assessment patterns when asked about their health. This is described as the reporting heterogeneity problem (Shmueli 2003). However, there is no reason to suspect a non-random distribution of measurement errors related to the PHQ-8, which is an instrument based on the DSM criteria for depression and has been applied in numerous countries and regions (making it possible to establish reasonable evidence in terms of its functioning). Its feasibility and accuracy make it a useful general population indicator (Pinto-Meza et al. 2005). The main cause of measurement errors in the data used in this study (from the EHIS) would be the use of proxy respondents. However, only 14 of the countries included permitted this use, which was confined to very specific situations in the other countries (described in the “Data and participants” section). Finally, it should be noted that the EHIS used compensation methods for non-response rates and therefore the potential non-response bias could be considered to be at least partially controlled (Eurostat 2018). Fourth, our study uses a national-scale measure of income inequality. However, it is necessary to take into account that different geographic scales (country, state/region, province, county, census tract, neighbourhood, etc.) are possible for this measurement. The available empirical evidence shows that there can be different interpretations or implications of societal characteristics in terms of mental health depending on the geographic scale that is used (Chen and Gotway Crawford 2012). It is important to take into account in this regard that the intensity of the role played by income inequality can differ depending on this circumstance. Research is required to provide clarification in this respect in the case of older adults in Europe.

## Conclusion

Promoting and protecting the mental health of older adults is a key factor in ensuring a successful ageing experience. Despite the limitations discussed, the findings of this study show the importance of the relationship between social support and socioeconomic inequalities measured at different and complementary levels. First, they highlight the importance of considering the increased inequality gaps affecting older adults in Europe as a risk factor for their mental health. Our findings in this respect indicate that more unequal societies provide a less favourable context for the mental health of older adults. In other words, the general/social context of inequality in which older adults are living plays a role in understanding symptoms of depression, in addition to merely inter-individual inequalities. Second, our results provide support for the hypothesis that the association between social support and depression varies significantly from country to country, suggesting that local contexts contribute distinctive and highly important elements in terms of understanding the relationship between social support and mental health among older adults. Both sets of findings are relevant for the development and testing of interventions designed to enhance mental health during the ageing process.

## Supplementary Information

Below is the link to the electronic supplementary material.

Supplementary file1 (DOCX 47 kb)

## Data Availability

Data are available under the rules established by Eurostat (https://ec.europa.eu/eurostat/web/microdata).

## Code availability (software application or custom code)

IBM-SPSS V.25 software was used.
