# Supplementary material for: Income inequalities, social support and depressive symptoms among older adults in Europe: a multilevel cross-sectional study
Source: Eur J Ageing. 2021 Dec 17;19(3):663–75. doi: 10.1007/s10433-021-00670-2 (PMC9424474; doi:10.1007/s10433-021-00670-2)
Supplement: Supplementary file 1 — Supplementary file1 (DOCX 47 kb) [file 10433_2021_670_MOESM1_ESM.docx]

Supplementary table 1. Participants main characteristics and sample distribution by country.

|  | | Female | Age  (reference “85+”) | | | | Living Alone | Activity limitation (reference “Not limited”) | | Education  (reference “Tertiary”) | | Equivalised household income (reference “Between 4^th^ and 5^th^“) | | | |  |
| --- | --- | --- | --- | --- | --- | --- | --- | --- | --- | --- | --- | --- | --- | --- | --- | --- |
|  | |  | 65-69 | 70-74 | 75-79 | 80-84 |  | Severely limited | Limited but not severely | Lower secondary or below | Upper secondary | Below first quintile | Between 1st and 2nd | Between 2nd and 3rd | Between 3rd and 4th | Subtotal |
| Austria | n | 1400 | 977 | 867 | 406 | 211 | 1805 | 311 | 904 | 726 | 1313 | 513 | 507 | 615 | 598 | 2561 |
|  | % | 54.7% | 38.1% | 33.9% | 15.9% | 8.2% | 70.5% | 12.1% | 35.3% | 28.3% | 51.3% | 20.0% | 19.8% | 24.0% | 23.4% | 100% |
| Bulgaria | n | 1068 | 603 | 479 | 316 | 239 | 1220 | 316 | 701 | 831 | 677 | 452 | 356 | 424 | 398 | 1758 |
|  | % | 60.1% | 34.0% | 27.0% | 17.8% | 13.5% | 68.7% | 17.8% | 39.6% | 46.8% | 38.1% | 25.7% | 20.3% | 24.1% | 22.6% | 100% |
| Czechia | n | 1575 | 778 | 638 | 448 | 374 | 1223 | 527 | 1126 | 561 | 1648 | 532 | 760 | 632 | 392 | 2442 |
|  | % | 63.8% | 31.5% | 25.9% | 18.2% | 15.2% | 49.6% | 21.4% | 45.6% | 22.7% | 66.8% | 21.8% | 31.1% | 25.9% | 16.1% | 100 % |
| Germany | n | 2891 | 1675 | 1690 | 1354 | 570 | 4055 | 709 | 1522 | 1538 | 2525 | 1266 | 1507 | 1257 | 922 | 5826 |
|  | % | 49.6% | 28.8% | 29.0% | 23.2% | 9.8% | 73.6% | 12.4% | 26.6% | 26.5% | 43.5% | 21.7% | 25.9% | 21.6% | 15.8% | 100% |
| Denmark | n | 924 | 604 | 554 | 310 | 189 | 1177 | 126 | 737 | 488 | 702 | 478 | 351 | 248 | 91 | 1262 |
|  | % | 51.2% | 33.5% | 30.7% | 17.2% | 10.5% | 67.2% | 7.2% | 41.9% | 28.3% | 40.7% | 37.9% | 27.8% | 19.7% | 7.2% | 100% |
| Estonia | n | 912 | 399 | 336 | 295 | 192 | 741 | 332 | 570 | 484 | 477 | 150 | 343 | 148 | 56 | 755 |
|  | % | 67.0% | 29.3% | 24.7% | 21.7% | 14.1% | 54.4% | 24.4% | 41.9% | 35.7% | 35.2% | 19.9% | 45.4% | 19.6% | 7.4% | 100% |
| Greece | n | 1643 | 696 | 568 | 624 | 483 | 1600 | 781 | 1030 | 2154 | 332 | 333 | 574 | 800 | 558 | 2699 |
|  | % | 60.9% | 25.8% | 21.0% | 23.1% | 17.9% | 59.3% | 29.0% | 38.3% | 79.8% | 12.3% | 12.3% | 21.3% | 29.6% | 20.7% | 100% |
| Finland | n | 1151 | 734 | 464 | 387 | 224 | 1212 | 183 | 812 | 867 | 534 | 504 | 564 | 383 | 258 | 1956 |
|  | % | 58.5% | 37.3% | 23.6% | 19.7% | 11.4% | 62.4% | 9.7% | 43.1% | 44.0% | 27.1% | 25.8% | 28.8% | 19.6% | 13.2% | 100% |
| France | n | 1857 | 1240 | 795 | 694 | 541 | 2872 | 615 | 1135 | 1889 | 1083 | 475 | 779 | 551 | 580 | 3015 |
|  | % | 51.9% | 34.6% | 22.2% | 19.4% | 15.1% | 80.2% | 17.6% | 32.4% | 53.7% | 30.8% | 15.8% | 25.8% | 18.3% | 19.2% | 100% |
| Hungary | n | 757 | 370 | 320 | 231 | 184 | 779 | 284 | 457 | 476 | 520 | 419 | 280 | 257 | 161 | 1216 |
|  | % | 62.3% | 30.4% | 26.3% | 19.0% | 15.1% | 64.3% | 23.4% | 37.6% | 39.1% | 42.8% | 34.5% | 23.0% | 21.1% | 13.2% | 100% |
| Ireland | n | 1603 | 955 | 754 | 582 | 396 | 1662 | 202 | 1163 | 1664 | 714 | 481 | 849 | 673 | 521 | 2918 |
|  | % | 54.9% | 32.7% | 25.8% | 19.9% | 13.6% | 57.7% | 7.5% | 43.3% | 57.8% | 24.8% | 16.5% | 29.1% | 23.1% | 17.9% | 100% |
| Italy | n | 3806 | 1900 | 1503 | 1368 | 1045 | 4855 | 1337 | 2321 | 5177 | 1159 | 846 | 1432 | 1641 | 1414 | 6798 |
|  | % | 56.0% | 27.9% | 22.1% | 20.1% | 15.4% | 71.4% | 20.1% | 35.0% | 76.2% | 17.0% | 12.4% | 21.1% | 24.1% | 20.8% | 100% |
| Lithuania | n | 987 | 385 | 355 | 354 | 200 | 850 | 337 | 761 | 658 | 587 | 229 | 557 | 461 | 172 | 1471 |
|  | % | 67.1% | 26.2% | 24.1% | 24.1% | 13.6% | 57.8% | 22.9% | 51.7% | 44.7% | 39.9% | 15.6% | 37.9% | 31.3% | 11.7% | 100% |
| Luxembourg | n | 298 | 229 | 178 | 128 | 87 | 430 | 89 | 252 | 307 | 209 | 56 | 100 | 139 | 95 | 474 |
|  | % | 45.2% | 34.7% | 27.0% | 19.4% | 13.2% | 68.6% | 14.1% | 40.0% | 49.0% | 33.4% | 11.8% | 21.1% | 29.3% | 20.0% | 100% |
| Latvia | n | 1350 | 512 | 523 | 448 | 300 | 1195 | 530 | 1003 | 677 | 927 | 419 | 692 | 532 | 232 | 1976 |
|  | % | 68.3% | 25.9% | 26.5% | 22.7% | 15.2% | 60.5% | 26.8% | 50.8% | 34.3% | 47.0% | 21.2% | 35.0% | 26.9% | 11.7% | 100% |
| Norway | n | 851 | 655 | 428 | 272 | 215 | 1143 | 155 | 215 | 392 | 889 | 301 | 478 | 371 | 327 | 1713 |
|  | % | 49.7% | 38.2% | 25.0% | 15.9% | 12.6% | 66.9% | 9.1% | 12.6% | 23.0% | 52.2% | 17.6% | 27.9% | 21.7% | 19.1% | 100.0% |
| Poland | n | 3359 | 1926 | 1243 | 1088 | 766 | 4098 | 1192 | 1959 | 2385 | 2546 | 604 | 1190 | 1288 | 1020 | 4743 |
|  | % | 60.7% | 34.8% | 22.4% | 19.6% | 13.8% | 74.0% | 21.6% | 35.4% | 43.1% | 46.0% | 12.7% | 25.1% | 27.2% | 21.5% | 100% |
| Portugal | n | 3486 | 1533 | 1319 | 1259 | 979 | 3371 | 1062 | 2415 | 5233 | 203 | 1368 | 1534 | 1174 | 867 | 5701 |
|  | % | 61.1% | 26.9% | 23.1% | 22.1% | 17.2% | 59.1% | 18.6% | 42.4% | 91.8% | 3.6% | 24.0% | 26.9% | 20.6% | 15.2% | 100% |
| Romania | n | 2556 | 1298 | 1108 | 1036 | 714 | 3102 | 482 | 1549 | 3012 | 1227 | 1048 | 1235 | 959 | 708 | 4485 |
|  | % | 57.0% | 28.9% | 24.7% | 23.1% | 15.9% | 69.2% | 10.7% | 34.5% | 67.2% | 27.4% | 23.4% | 27.5% | 21.4% | 15.8% | 100% |
| Sweden | n | 680 | 419 | 350 | 248 | 164 | 909 | 141 | 476 | 517 | 454 | 109 | 560 | 306 | 165 | 1302 |
|  | % | 52.2% | 32.2% | 26.9% | 19.0% | 12.6% | 69.8% | 11.1% | 37.4% | 40.1% | 35.2% | 8.4% | 43.0% | 23.5% | 12.7% | 100% |
| Slovenia | n | 829 | 441 | 374 | 271 | 221 | 1021 | 268 | 640 | 513 | 701 | 345 | 314 | 204 | 120 | 1065 |
|  | % | 57.7% | 30.7% | 26.0% | 18.9% | 15.4% | 71.2% | 18.7% | 44.7% | 36.0% | 49.2% | 32.4% | 29.5% | 19.2% | 11.3% | 100% |
| Slovakia | n | 738 | 382 | 310 | 253 | 220 | 617 | 322 | 580 | 356 | 682 | 85 | 410 | 448 | 137 | 1141 |
|  | % | 63.3% | 32.8% | 26.6% | 21.7% | 18.9% | 53.0% | 27.6% | 49.8% | 30.6% | 58.5% | 7.4% | 35.9% | 39.3% | 12.0% | 100% |
| UK | n | 3827 | 2466 | 1853 | 1434 | 869 | 4694 | 930 | 2545 | 2905 | 2407 | 1499 | 1711 | 1723 | 1258 | 7186 |
|  | % | 53.3% | 34.3% | 25.8% | 20.0% | 12.1% | 65.3% | 13.0% | 35.5% | 40.9% | 33.9% | 20.9% | 23.8% | 24.0% | 17.5% | 100% |
| Iceland | n | 403 | 254 | 211 | 341 | 0 | 550 | 182 | 121 | 363 | 313 | 202 | 217 | 140 | 112 | 806 |
|  | % | 50.0% | 31.5% | 26.2% | 42.3% | 0.0% | 68.2% | 22.8% | 15.2% | 45.4% | 39.1% | 25.1% | 26.9% | 17.4% | 13.9% | 100% |
| Total | n | 38951 | 21431 | 17220 | 14147 | 9383 | 45181 | 11413 | 24994 | 34173 | 22829 | 12714 | 17300 | 15374 | 11162 | 65269 |
|  | % | 56.9% | 31.3% | 25.2% | 20.7% | 13.7% | 66.5% | 16.9% | 37.0% | 50.2% | 33.6% | 19.5% | 26.5% | 23.6% | 17.1% | 100% |

Supplementary table 2. Robustness checks for model 4. Bootstrap analysis and separate model fit for men and women^a^.

| **Variable** | **Lvl** | **Bootstrap analysis (n=100)** | **Men** | **Women** |
| --- | --- | --- | --- | --- |
| **Fixed effects** |  |  |  |  |
| Intercept | 1 | 2.21^**^ (.04) [2.11,2.30] | 2.11^***^ (.21) [1.70,2.53] | 1.61^***^ (.14) [1.31,1.92] |
| Age | 1 | .08^**^ (.02) [.04,.13] | .05 (.04) [-.03,.14] | .12^**^ (.04) [.03,.21] |
| Female | 1 | -.69^**^ (.02) [-.73,-.64] |  |  |
| Living Alone | 1 | .12^**^  (.02) [.06,.17] | .09^*^  (.03) [.008,.18] | .21^***^ (.05) [.11,.31] |
| GALI | 1 |  |  |  |
| *1 (Limited but not severely)* |  | 3.28^**^  (.05) [3.17,3.38] | 3.38^***^  (.07) [3.24,3.53] | 3.04^***^  (.07) [2.89,3.20] |
| *2 (Severely limited)* |  | 1.40^**^ (.02) [1.35,1.44] | 1.49^***^ (.04) [1.39,1.58] | 1.19^***^ (.04) [1.10,1.28] |
| ADL | 1 | .75^**^ (.01) [.73,.78] | .71^***^ (.01) [.69,.73] | .84^***^ (.01) [.81,.86] |
| Education (ref. University) | 1 |  |  |  |
| *Compulsory* |  | .19^**^ (.03) [.13,.26] | .27^***^ (.07) [.13,.41] | .11 (.06) [-.01,.24] |
| *Secondary* |  | -.01 (.02) [-.06,.04] | -.03 (.07) [-.18,.10] | .05 (.05) [-.05,.16] |
| Income Quintile (ref. Q5, highest income*)* | 1 |  |  |  |
| *Q1* |  | .37^***^ (.04) [.27,.46] | .43^***^ (.08) [.27,.60] | .30^***^ (.08) [.14,.46] |
| *Q2* |  | .24^***^ (.04) [.14,.33] | .31^***^ (.08) [.15,.46] | .15^*^ (.07) [.01,.29] |
| *Q3* |  | .22^***^ (.04) [.13,.30] | .25^**^ (.08) [.09,.41] | .19^**^ (.06) [.05,.32] |
| *Q4* |  | .16^**^  (.04) [.08,.26] | .26^**^  (.08) [.09,.43] | .07 (.07) [-.06,.21] |
| Social Support | 1 | -.16^**^  (.01) [-.19,-.12] | -.20^***^  (.03) [-.26,-.13] | -.12^***^  (.02) [-.17,-.06] |
|  |  |  |  |  |
| Gini Coefficient | 2 | 11.10^*^ (2.30) [6.93,16.29] | 12.97^*^ (5.79) [1.61,24.33] | 8.56 (5.23) [-2.41,19.53] |
| Dependency ratio | 2 | -.02 (.01) [-.07,.006] | -.01 (.04) [-.09,.07] | -.06 (.03) [-.14,.01] |
| Healthcare expenditure | 2 | .17 (.03) [.10,.23] | .19^*^ (.08) [.02,.36] | .09 (.07) [-.07,.26] |
| **Interactions** |  |  |  |  |
| Social Support * Income Quintile | 1x1 |  |  |  |
| *Q1* |  | -.11^**^ (.02) [-.14,-.06] | -.09^**^ (.03) [-.17,-.02] | -.11^***^ (.02) [-.18,-.04] |
| *Q2* |  | -.07^**^ (.02) [-.12,-.04] | -.05 (.03) [-.13,.01] | -.09^**^ (.03) [-.15,-.02] |
| *Q3* |  | -.01 (.02) [-.05,.02] | -.007 (.03) [-.08,.06] | -.007 (.03) [-.07,.05] |
| *Q4* |  | -.01 (.02) [-.07,.01] | -.001 (.04) [-.08,.08] | -.03 (.03) [-.10,.03] |
| **Random Effects** |  |  |  |  |
| Residual |  | 11.01^**^ (.09) [10.83,11.20] | 12.77^***^ (.10) [12.56,12.98] | 8.12^***^ (.08) [7.95,8.28] |
| Intercept |  | .63^**^ (.08) [.55,.89] | .81^**^ (.27) [.42,1.56] | .36^**^ (.12) [.19,.71] |
| Social Support |  | -.03^*^(.01) [-.06,-.02] | -.04^*^ (.01) [-.08,-.01] | -.01 (.01) [-.03,.002] |
| Intercept*Social Support |  | .002^*^(.001) [.0008, .004] | .001 (.001) [.0003,.02] | .001 (.001) [.0003, .008] |
| **Model fit** |  |  |  |  |
| Log.likelihood -2 |  | 246008.19 | 153884.03 | 90870.99 |
| Akaike (AIC) |  | 246016.19 | 153892.03 | 90878.99 |

^a^ Format: Multilevel coefficient (standard error) [95% confidence interval]. ^***^ p<.001; ^**^ p<.01; ^*^ p<.05.

^***^ p<.001; ^**^ p<.01; ^*^ p<.05
